# Supplementary material for: Mathematical Modelling Based on In Vivo Imaging Suggests CD137-Stimulated Cytotoxic T Lymphocytes Exert Superior Tumour Control Due to an Enhanced Antimitotic Effect on Tumour Cells
Source: Cancers (Basel). 2021 May 24;13(11):2567. doi: 10.3390/cancers13112567 (PMC8197176; doi:10.3390/cancers13112567)
Supplement: Supplementary file 1 [file cancers-13-02567-s001.zip › cancers-1192769-supplementary.pdf]

Mathematical Modelling Based on In Vivo Imaging Suggests CD137-Stimulated Cytotoxic T Lymphocytes Exert Superior Tumour Control Due to an Enhanced Antimitotic Effect on Tumour Cells

Richard J. Beck, Bettina Weigelin, Joost B. Beltman

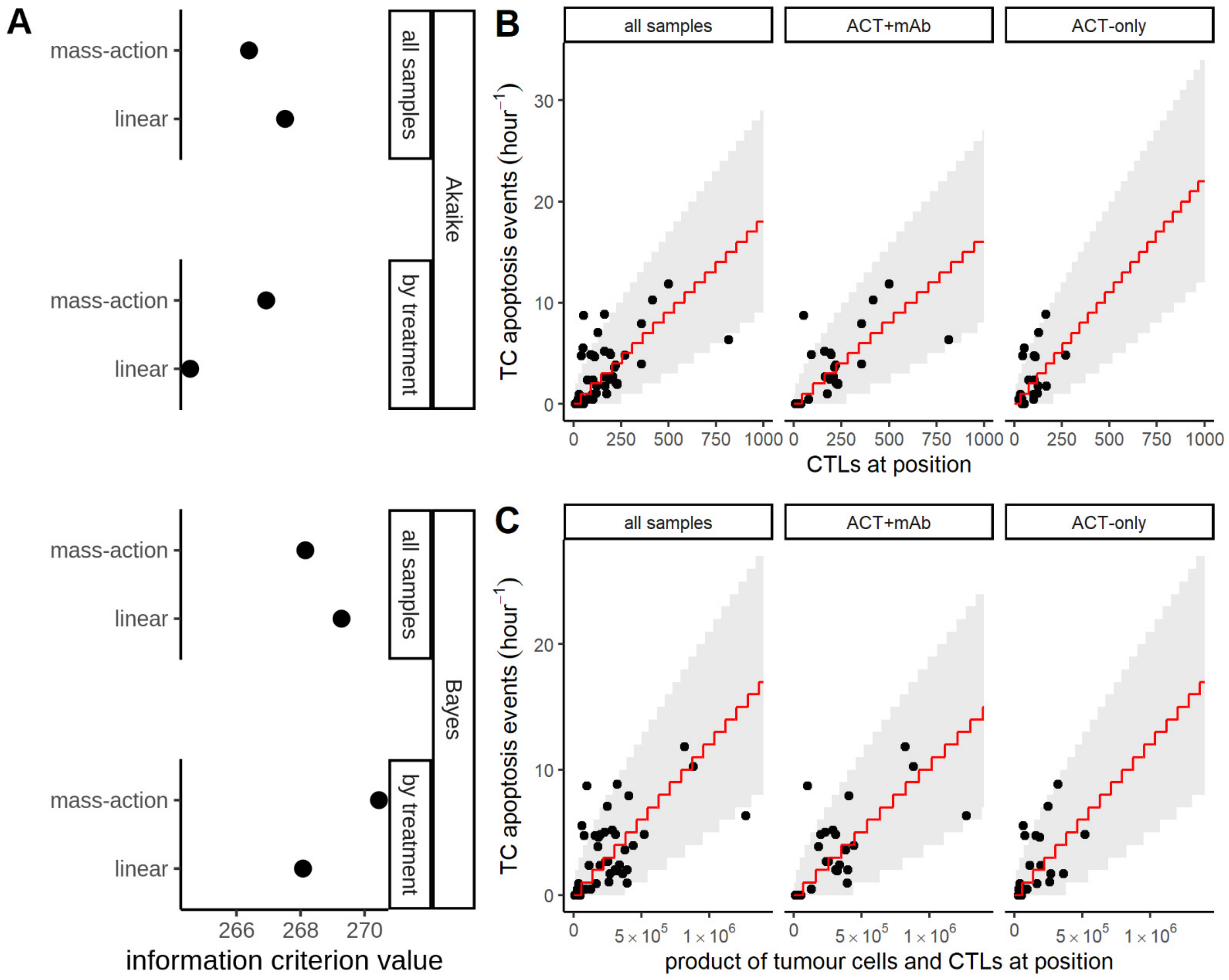

Figure S1. Information content of linear or mass-action CTL killing models. (**A**) Akaike (top 2 rows) or Bayes (bottom 2 rows) information criteria values for either linear or mass-action killing models. For the linear model, the killing intensity is taken to be linearly proportional to the number of CTLs. For the mass-action killing model, the killing intensity is proportional to the product of the number of CTLs and tumour cells at each position. Fits were performed for either all the samples grouped together (rows 1&3), or separately for ACT+mAb and ACT-only treatment conditions (rows 2&4). (**B**–**C**) Linear killing (**B**) or mass action killing (**C**) model fits for all samples grouped together (left columns), or ACT-only (central columns) and ACT+mAb (right columns) fitted separately.

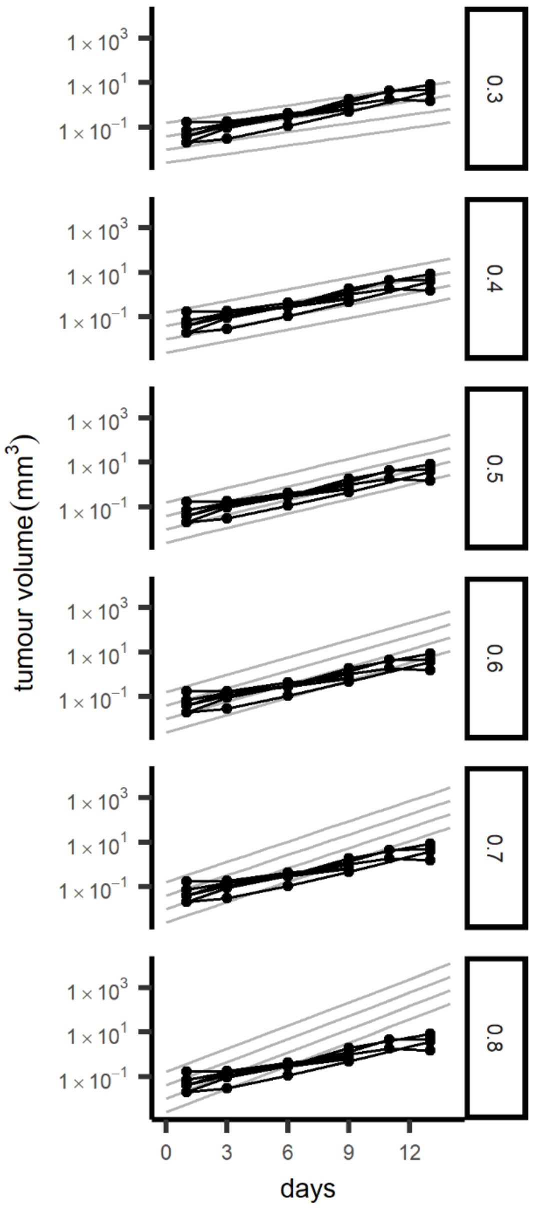

Figure S2. Compatibility of different growth rates with untreated tumour data. Black lines and points show the volume progression of OVA⁺ tumours which did not receive ACT. Grey lines represent exponential growth for different values of the growth rate parameter (g) as indicated for each row.

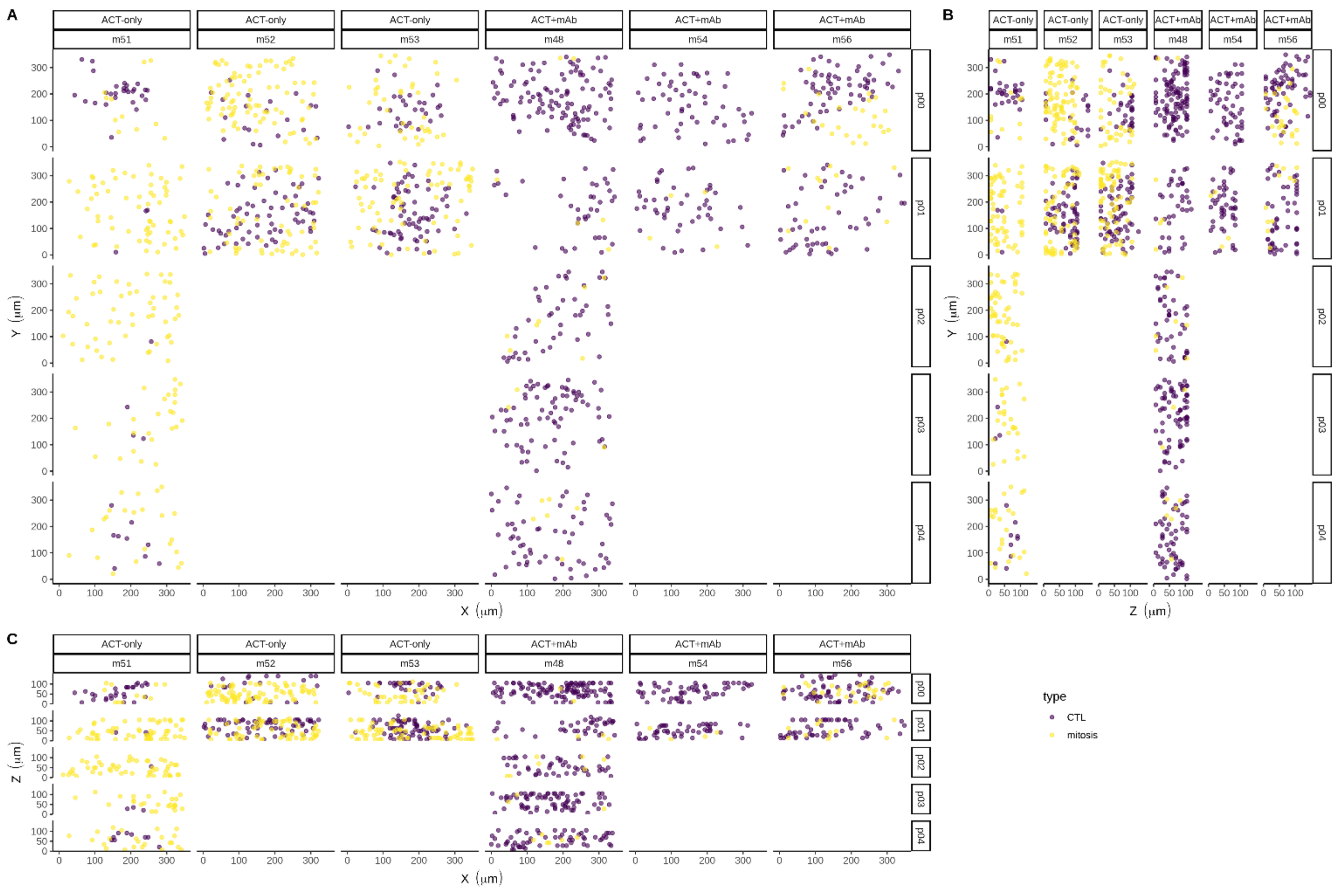

Figure S3. Quantification of CTLs and TC mitosis in day 7 treated data. (**A**–**C**) Purple dots are the locations of individual CTLs in the first frame, and yellow dots are the locations of mitotic events (only frames 1–30 were counted, = 1hour). Each image shows all points counted per position, thus A-C are three "max intensity" style views of a single point cloud per position (rows) and mouse (columns), i.e. views of the xy axes (**A**), yz axes (**B**) and xz axes (**C**). XYZ (z = slice) axes are shown with a fixed scaling relative to their length – 0.7 pixels (xy) = 1um, 1 slice = 7um. All points were determined manually.

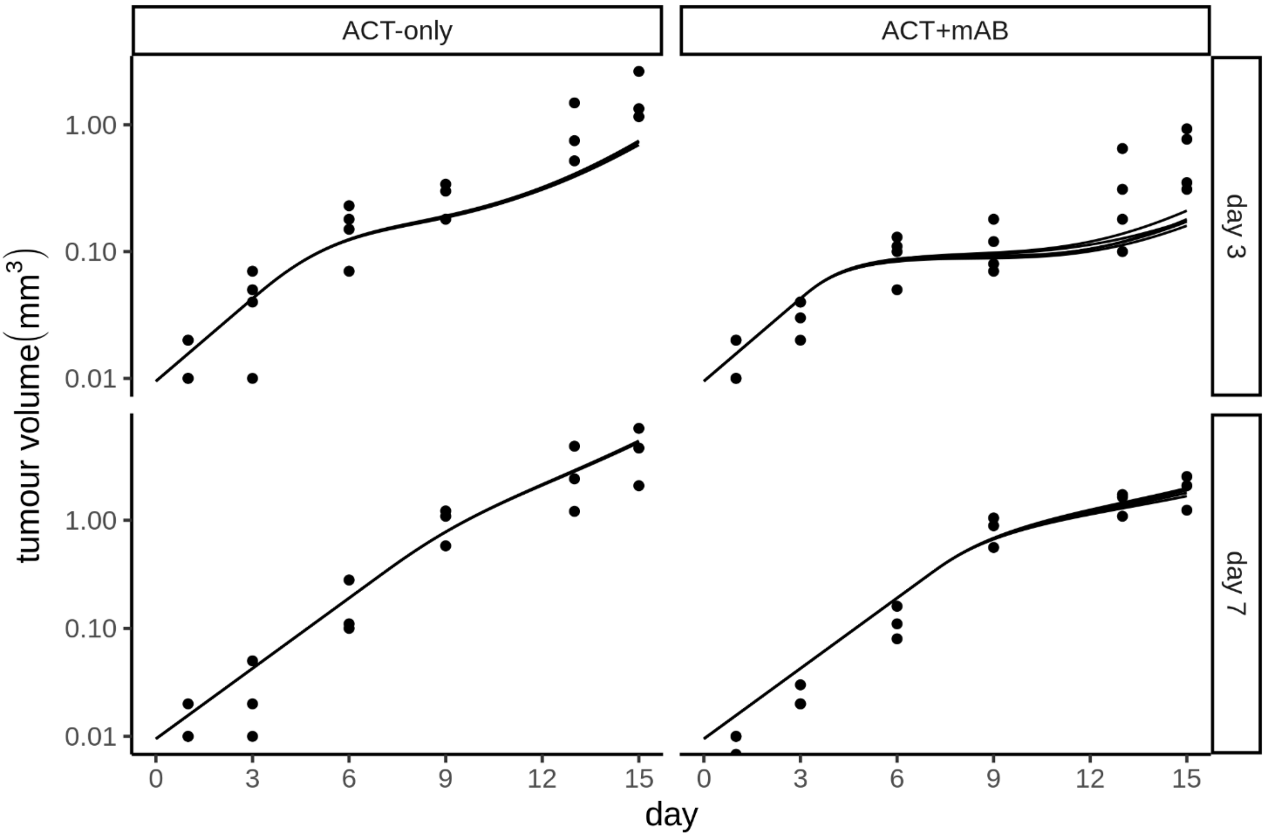

Figure S4. Verification of model fit with tumour volume progression data. Lines indicate model output and points are experimental measurements. Model comparison is with ACT-only (left column) or ACT+mAb (right column), when ACT was given on either day 3 (top row) or day 7 (bottom row). To generate this comparison, the mean number of tumour cells per position ($\bar{n}_p$) in the intravital data was calculated. Then, $\rho = {}^{\bar{n}_p}/{}_{V_p}$ is an estimate of the spatial density of tumour cells, where V_p is the volume imaged per position. After simulating the models with the best fitting parameters, we multiplied the total number of tumour cells in the model by ρ to convert from number of cells to volume.

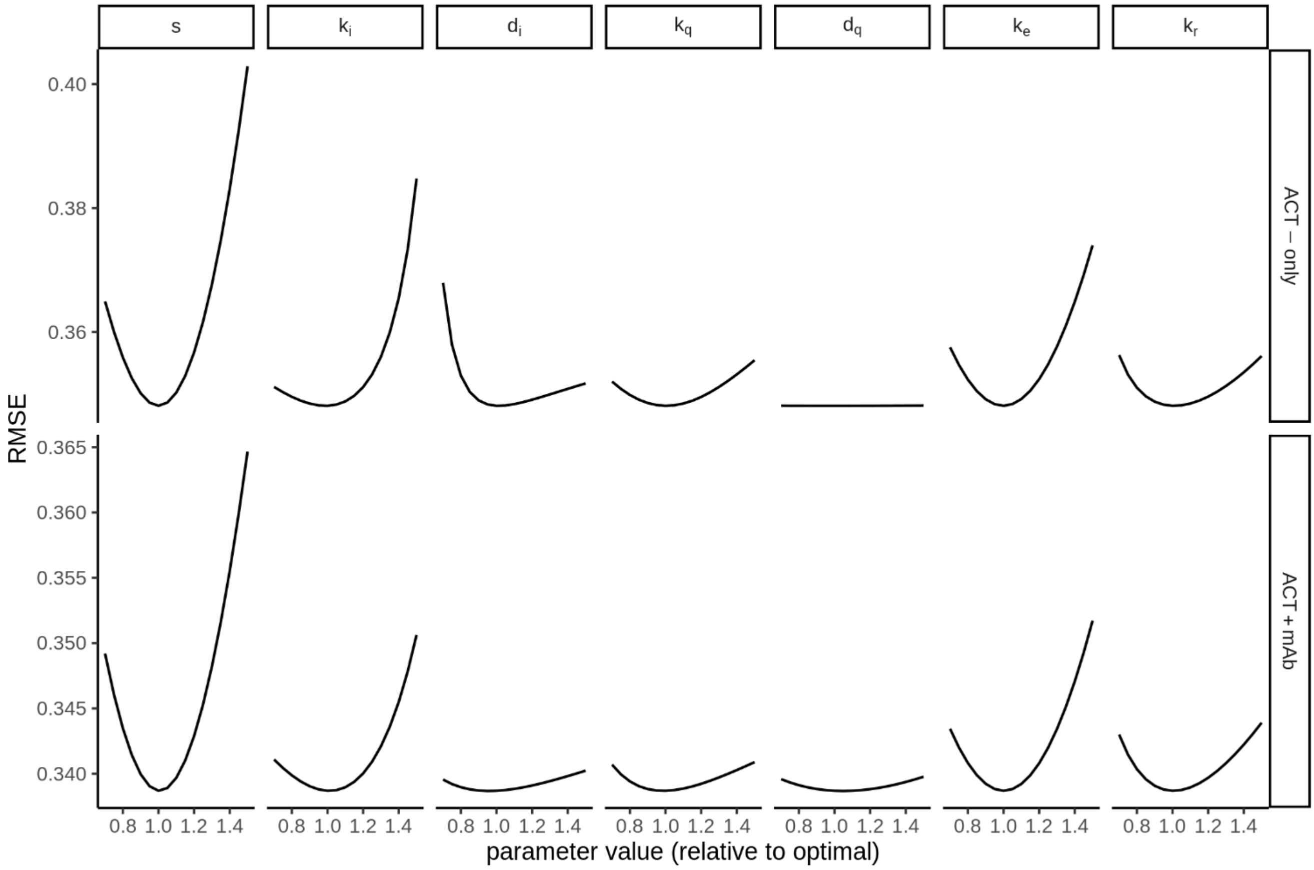

Figure S5. Local sensitivity analysis of model parameters. Starting with the best fitting model parameters for the ACT-only (top row) or ACT+mAb (bottom row) conditions, we varied each parameter individually (across columns) and quantified the RMSE (y axis) associated with varying each parameter relative to its optimal value.

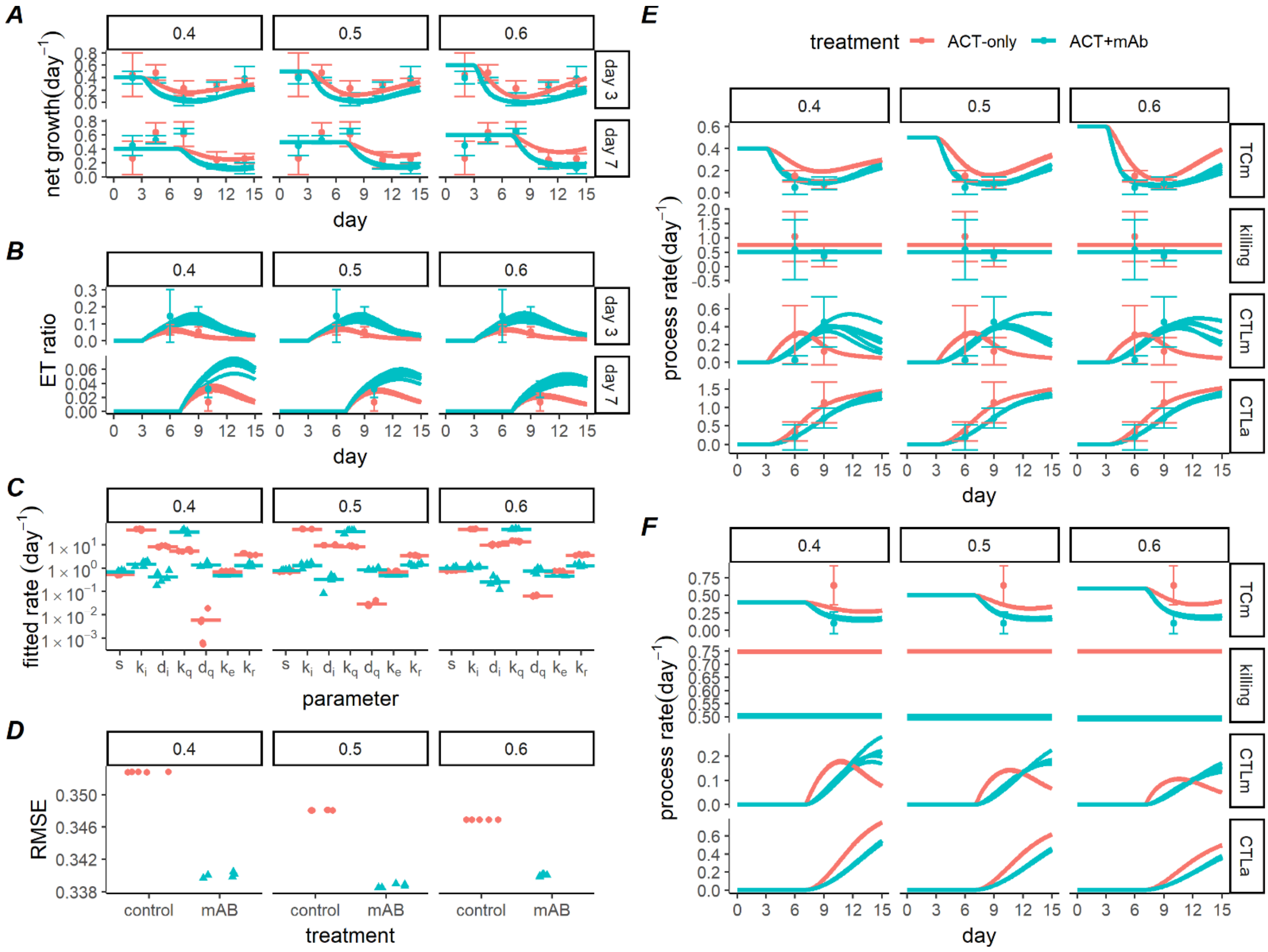

Figure S6. Sensitivity of ODE model fitting to the growth rate parameter, g. For all panels the preset growth rate parameter is indicated by the value across columns. (**A**) Net tumour growth predicted by each of the best fitting parameter sets, for either tumours exposed to ACT on day 3 (top row) or day 7 (bottom row). (**B**) Effector:target ratio predicted by each of the best fitting parameter sets, for either tumours exposed to ACT on day 3 (top row) or day 7 (bottom row). (**C**) Best fitting parameters for the ODE model. Each point represents 1 of 5 fits using the stochastic evolutionary algorithm, and horizontal lines represent the mean fitted parameter for either ACT-only (red) or ACT+mAb (blue) conditions. (**D**) Root Mean Square Error (RMSE) for each fitted parameter set. (**E**–**F**) Intravital process rates predicted by each of the best fitting parameter sets for tumours exposed to ACT on day 3 (**E**) or day 7 (**F**). Process rates considered are: TCm (Tumour Cell mitosis); killing (of tumour cells by CTLs); CTLm (CTL mitosis); CTLa (CTL apoptosis).
